# Aging-related predictive factors for oxygenation improvement and mortality in COVID-19 and acute respiratory distress syndrome (ARDS) patients exposed to prone position: A multicenter cohort study

**DOI:** 10.1016/j.clinsp.2023.100180

**Published:** 2023-03-09

**Authors:** Marieta C.A. Cunha, Jociane Schardong, Natiele C. Righi, Adriana C. Lunardi, Guadalupe N. Sant'Anna, Larissa P. Isensee, Rafaela F. Xavier, Jose E. Pompeu, Ranata M. Weigert, Darlan L. Matte, Rozana A. Cardoso, Ana C.V. Abras, Antonio M.V. Silva, Camila C. Dorneles, Roberta W. Werle, Ana C. Starke, Juliana C. Ferreira, Rodrigo D.M. Plentz, Celso R.F. Carvalho

**Affiliations:** a Pulmonary Division, Heart Institute, Faculdade de Medicina da Universidade de São Paulo, São Paulo, SP, Brazil; b Physiotherapy Service, Irmandade Santa Casa de Misericórdia de Porto Alegre, Universidade Federal de Ciências da Saúde de Porto Alegre, Porto Alegre, RS, Brazil; c Department of Physical Therapy, Faculdade de Medicina da Universidade de São Paulo, São Paulo, SP, Brazil; d Department of Physical Therapy, Tacchini Hospital, Bento Gonçalves, RS, Brazil; e Department of Neuroscience, Universidade do Estado de Santa Catarina, Florianópolis, SC, Brazil; f Department of Physical Therapy, Santa Casa de Misericórdia de Belo Horizonte, Belo Horizonte, MG, Brazil; g Department of Physiotherapy and Rehabilitation, Universidade Federal de Santa Maria, Santa Maria, RS, Brazil; h Santa Maria University Hospital, Universidade Federal de Santa Maria (UFSM), Santa Maria, RS, Brazil; i University Hospital Polydoro Ernani de São Thiago, Universidade Federal de Santa Catarina, Florianópolis, SC, Brazil

**Keywords:** Acute respiratory distress syndrome, Adult respiratory distress syndrome, Coronavirus infections, COVID-19, Prone position

## Abstract

**Background:**

Elderly patients are more susceptible to Coronavirus Disease-2019 (COVID-19) and are more likely to develop it in severe forms, (e.g., Acute Respiratory Distress Syndrome [ARDS]). Prone positioning is a treatment strategy for severe ARDS; however, its response in the elderly population remains poorly understood. The main objective was to evaluate the predictive response and mortality of elderly patients exposed to prone positioning due to ARDS-COVID-19.

**Methods:**

This retrospective multicenter cohort study involved 223 patients aged ≥ 65 years, who received prone position sessions for severe ARDS due to COVID-19, using invasive mechanical ventilation. The PaO_2_/FiO_2_ ratio was used to assess the oxygenation response. The 20-point improvement in PaO_2_/FiO_2_ after the first prone session was considered for good response. Data were collected from electronic medical records, including demographic data, laboratory/image exams, complications, comorbidities, SAPS III and SOFA scores, use of anticoagulants and vasopressors, ventilator settings, and respiratory system mechanics. Mortality was defined as deaths that occurred until hospital discharge.

**Results:**

Most patients were male, with arterial hypertension and diabetes mellitus as the most prevalent comorbidities. The *non-responders* group had higher SAPS III and SOFA scores, and a higher incidence of complications. There was no difference in mortality rate. A lower SAPS III score was a predictor of oxygenation response, and the male sex was a risk predictor of mortality.

**Conclusion:**

The present study suggests the oxygenation response to prone positioning in elderly patients with severe COVID-19-ARDS correlates with the SAPS III score. Furthermore, the male sex is a risk predictor of mortality.

## Highlights

• Prone position in intubated patients with COVID-19 improves gas exchange.
• Elderly and severe comorbidities increase mortality risk after prone sessions for ARDS-COVID-19.
• ARDS-COVID-19 better respond when prone is applied early in patients with good health status.

## Introduction

Viral pneumonia is the most severe manifestation caused by the novel coronavirus, leading to acute respiratory distress syndrome related to COVID-19 disease (COVID-19-ARDS).^1^, ^2^, ^3^, ^4^ COVID-19-ARDS is diagnosed by confirming SARS-CoV-2 infection and the presence of ARDS signs classified according to Berlin criteria (2012).^1^, ^2^, ^3^, ^4^

COVID-19-ARDS is caused by an exacerbated increase in proinflammatory cytokines and other inflammatory markers, known as a cytokine storm. The inflammatory reaction causes diffuse alveolar damage and hyaline membrane formation in the alveoli, generating edema and fibroblast proliferation.^4^ Associated with the inflammatory exacerbated reaction, COVID-19-ARDS presents coagulation dysfunction, detected by high levels of D-dimer. This association may explain the atypical manifestations found in patients with COVID-19, such as dilatation of the pulmonary vessels, which is rarely found in patients with classic ARDS.^4^

The prone position is considered an adjunct treatment for intubated patients with severe COVID-19-ARDS since the Surviving Sepsis Campaign and the World Health Organization recommendations.^5^^,^^6^ Using the prone position is well known to improve oxygenation and reduce the risk of mortality in classic ARDS with refractory hypoxemia.^5^, ^6^, ^7^, ^8^, ^9^ Better outcomes are achieved if the prone position is applied in the first 48h and at least for 12‒16h. Additionally, it may be associated with protective ventilatory strategies, neuromuscular blockade, and permissive hypercapnia.^3^^,^^5^^,^^6^^,^^10^, ^11^, ^12^

Lung protective ventilation plays important role in improving prone outcomes. It is recommended that the patients be ventilated with low tidal volumes (4–8 mL/kg of predicted body weight), low plateau pressures (< 30–32 cm H_2_O.), and driving pressures below 14‒15 cm H_2_O.^13^ The PaO_2_/FiO_2_ is used to assess the oxygenation response in patients with ARDS. Although the cutoff value has not been well established, most studies use improvement cutoff values of 10‒20 mmHg PaO_2_ or PaO_2_/FiO_2_ or a 10%‒20% increase in PaO_2_/FiO_2_.^13^^,^^14^

Several studies have shown that older adults present the most severe form of the disease and high mortality rate. Evidence suggests that advanced age is the most important predictor of mortality, especially among adults aged > 80 years.^15^^,^^16^ Advanced age causes progressive lung function impairment due to structural changes that impaired gas exchange and immunological changes, predisposing to infections. Molecular and immunological changes may explain why elderly patients have a worse prognosis with COVID-19.^15^^,^^16^

In healthy aging, lung reserve is naturally reduced. The lung in the elderly is characterized by a lower density of bronchioles and an increase in their diameter. There is a loss of alveolar surface area and an increase in the size of the alveoli and airspace. In addition, there is a reduction in lung elasticity, making it more rigid. It is expected that the lungs of the elderly have a greater functional residual capacity and a lower forced expiratory volume in the 1^st^ second/forced vital capacity ratio (FEV_1_/FVC). Also, both FEV_1_ and FVC are lower with advanced age.^17^

Due to the frailty of elderly patients, it is mandatory to understand the response to prone positioning for ARDS due to COVID-19. Understanding this treatment's effectiveness may result in more humanized care and therapeutic proportionality. However, there is a lack of understanding of oxygenation improvement and mortality risk after a prone position in this population. Therefore, the primary objective of this study was to identify predictors of oxygenation response and mortality risk after prone positioning in elderly patients with severe COVID-19-ARDS. The secondary objective was to assess the response to prone positioning in elderly patients who developed the most severe form of the disease.

## Methods

This multicenter retrospective cohort study was conducted in six hospitals and approved by the Clinical Research Ethics Committee of all centers (31881520.3.1001.5335). Due to the retrospective nature of the study, the need for informed consent was waived. The study included patients under invasive mechanical ventilation with suspected or confirmed SARS-CoV-2 infection, who received prone position sessions for severe COVID-19-ARDS treatment. The inclusion criteria were individuals diagnosed with COVID-19, requiring invasive mechanical ventilation and severe ARDS (PaO_2_/FiO_2_ < 150 mmHg). The exclusion criterion was age < 65 years.

Confirmed COVID-19 patients were considered for analysis if they presented a positive real-time reverse transcription-polymerase chain reaction (PCR-RT). Additionally, patients with suspected or negative PCR-RT who presented clinical symptoms of COVID-19, including fever, cough, tiredness, anosmia, ageusia, headache, pain, diarrhea, and/or dyspnea, were also included.

The trained researchers collected data from electronic medical records using standardized forms. All contributors had access to the electronic medical records of their affiliated institutions and were committed to ensuring data protection. Patients were followed up from hospital admission to discharge or death, and the study group did not interfere with medical decisions.

The PaO_2_/FiO_2_ ratio was used to assess the oxygenation response. Patients who presented a 20-point improvement in PaO_2_/FiO_2_ after the first prone session were considered the *responders* group. Patients who did not present 20-point of improvement in PaO_2_/FiO_2_ after the first prone session were included in the *non-responders* group. Mortality was defined as deaths that occurred between hospitalization and discharge.

### Data collection

The following data were collected: demographic information, comorbidities, complications, D-dimer level, Simplified Acute Physiology Score (SAPS III), Sequential Organ Failure Assessment (SOFA) score, PaO_2_/FiO_2_ ratio, Body Mass Index (BMI), comorbidities, and use of anticoagulants and vasopressors. SAPS III and SOFA scores considered for analysis were calculated at the Intensive Care Unit (ICU) admission. D-dimer levels were evaluated using the HemosIL HS-500 automated immunoassay (HemosIL® D-dimer HS 500, Instrumentation Laboratory, 80003610270, Instrumental Laboratory Company, Bedford, MA, USA).

Comorbidities were assessed, including immunosuppression, arterial hypertension, diabetes, obesity, smoking, alcohol consumption, and neurological, hematological, respiratory, and cardiovascular diseases. Furthermore, immunosuppression was defined as a history of organ transplantation, chronic kidney disease, HIV infection, AIDS, and cancer treatment.

Clinical data included arterial blood gas analysis before and after the first prone session. In addition, the time until the first prone positioning, duration of the first prone session (in hours), number of prone sessions, and complications related to prone positioning were also collected. The time between the first intubation and the prone session was considered the first prone position. Unfortunately, due to hospital bed overload, it was impossible to collect data for blood gas analysis from the health staff on time. Therefore, the data considered for the analysis were obtained closest to the beginning and end of the first prone session.

Ventilator settings and respiratory mechanics calculations, such as Driving Pressure (DP), Plateau Pressure (Pplat), and respiratory system static Compliance (Cst), were collected before and after the first prone session. The total duration of the first prone session and a number of prone cycles were recorded. Furthermore, adverse effects, such as decreased oxygenation level, accidental extubation, central venous or arterial line removal, hemodynamic instability, acute arrhythmia, cardiopulmonary arrest, and vomiting, were recorded. Patient outcomes, including duration of invasive mechanical ventilation, length of hospital and ICU stay, reintubation, and survival, were also recorded.

### Statistical analysis

The sample size was calculated considering a 25% mortality and including at least five independent variables. A sample calculation was performed according to the following formula: (10*[k+1]), where *k* represents the number of explanatory variables of the predictive model.^18^ A total of 60 patients was calculated. Continuous variables were expressed as medians and 25%−75% interquartile ranges, while categorical variables were expressed as the number of patients and percentages. Between-group comparisons were performed using the Mann-Whitney test. Logistic regression was used to evaluate the factors associated with the response to prone positioning and mortality. Variables with a p-value < 0.2 were used for multivariate regression. Finally, multicollinearity was assessed by examining variance inflation factors. The results are presented as Odds Ratios (OR) with a 95% Confidence Interval (CI). The IBM SPSS Statistics package (Version 26.0) was used for statistical analysis, and a p-value < 0.05 was set as significant.

## Results

The study included 223 patients; the patients were divided into two groups according to an increase in PaO_2_/FiO_2_ (*responders* [72.6%] and *non-responders* [27.3%]). The average age of the patients was 72 (68−76) years, most patients were male (60.1%), and the most prevalent comorbidities were hypertension, diabetes mellitus, and obesity (Table 1).

**Table 1:** Baseline characteristics of patients with COVID-19 and ARDS at ICU admission.

Outcomes	All patients(n = 223)	*Responders*(n = 162)	*Non-responders*(n = 61)	p-value
**Anthropometric data**				
Age (y/o)	72 (68‒76)	71 (67‒76)	73 (69‒77)	0.25
Male sex, n (%)	134 (60.1)	96 (59.3)	38 (62.3)	0.68
BMI (kg/m²)	27.7 (25‒31)	27.6 (25‒31)	28.2 (25‒31)	0.37
**Comorbidities**				
AH, n (%)	164 (73.5)	117 (72.2)	47 (77)	0.46
DM, n (%)	102 (45.9)	74 (45.7)	28 (46.7)	0.89
Obesity, n (%)	72 (32.6)	52 (32.1)	20 (33.9)	0.80
Smoker, n (%)	52 (23.5)	37 (22.8)	15 (25.4)	0.69
CPD, n (%)	32 (14.3)	24 (14.8)	8 (13.3)	0.78
Immunosuppression, n (%)	29 (13)	20 (12.3)	9 (15.3)	0.57
**SAPS III (score)**	73 (59‒81)	72 (56‒79)	78 (66‒86)	**0.011**
**SOFA (score)**	10 (7‒13)	9 (6‒12)	11 (9‒14)	**0.018**
**D-dimer (ng/mL)**	2.80 (949‒5.79)	1.86 (865‒5.34)	2.45 (1.35‒7.53)	0.47

Data are presented as the average and interquartile range (25%‒75%) or the number of subjects and percentage, in parenthesis.

BMI, Body Mass Index; AH, Arterial Hypertension; DM, Diabetes Mellitus; CPD, Chronic Pulmonary Disease; SAPS III, Simplified Acute Physiology Score; SOFA, Sequential Organ Failure Assessment. Clinical data were evaluated, and there was no interference by the investigators.

Both groups presented similar anthropometric characteristics, such as age, sex, BMI, comorbidities, and D-dimer levels. However, higher SAPS III and SOFA scores were observed in the non-responder group, indicating an independent mortality risk between the two groups (Table 1).

A protective ventilatory strategy was applied in both groups, following the guideline recommendations for low DP and Pplat. The average DP was 12 cm H_2_O, and the average respiratory Cst was 31 mL/cm H_2_O. No differences were observed between DP and respiratory Cst between the two groups (Table 2). Furthermore, the ventilatory settings were similar between the groups, except for a lower FiO_2_ at baseline found in the *non-responders* group (p = 0.05).

**Table 2:** Ventilator settings, prone position response, and patient outcomes.

Outcomes	All patients(n = 223)	*Responders*(n = 162)	*Non-responders*(n = 61)	p-value
**Pre-prone mechanical ventilation**				
PEEP (cm H_2_O)	10 (9‒12)	10 (10‒12)	10 (9‒12)	0.35
FiO_2_ (%)	80 (65‒100)	80 (65‒100)	70 (60‒90)	**0.05**
RR (bpm)	28 (23‒32)	26 (22‒31)	30 (25‒33)	0.47
Tidal volume (mL)	390 (340‒440)	389 (329‒442)	390 (345‒420)	0.62
Driving pressure (cm H_2_O)	12 (10‒15)	12 (10‒15)	13 (10‒15)	0.84
Pplat (cm H_2_O)	24 (21‒26)	24 (21‒27)	23 (20‒25)	0.47
Cst (mL/cm H_2_O)	31 (24‒39)	31 (24‒38)	31.5 (27‒39)	0.46
**Pre prone blood gases analysis**				
Arterial Ph	7.32 (7.25‒7.38)	7.30 (7.30‒7.40)	7.32 (7.24‒7.38)	0.09
PaO_2_ (mmHg)	72 (63.5‒81)	72 (63‒82)	71 (63.5‒81)	0.78
PaCO_2_ (mmHg)	51.7 (45‒61)	52 (45‒61)	51.5 (45.1‒61.6)	0.58
HCO_3_ (mEq/L)	26.3 (22‒30)	26.5 (22‒31)	25.4 (22.1‒28.4)	0.43
Initial PaO_2_/FiO_2_ (mm Hg)	95 (79‒120)	93 (77‒118)	106 (82‒120)	0.16
Δ PaO_2_/FiO_2_ (mmHg)	43 (15‒80)	62 (39‒101)	0.5 (-10.1‒8.2)	**< 0.001**
Δ PCO_2_ (mmHg)	-0.9 (‒10‒5)	-0.9 (-10‒6)	-0.9 (-10‒3.4)	0.75
**Complications**	15 (6.7)	3 (1.9)	12 (19.7)	**< 0.001**
**Drug**				
ACO, n (%)	219 (98.6)	158 (98.1)	61 (100)	0.29
Vasopressors, n (%)	205 (91.9)	147 (90.7)	58 (95.1)	0.28
**Outcomes**				
Time until 1^st^ prone, days	2 (1‒6)	2 (1‒6)	3 (2‒6)	0.15
Duration of 1^st^ prone, hours	18 (16.2‒20)	18 (16.5‒20)	17.4 (15.6‒19.7)	0.09
Prone sessions, no. sessions	2 (1‒3)	2 (1‒3)	2 (1‒2)	0.39
Duration of IMV, days	15 (9‒22)	15 (9‒22)	16 (10‒24)	0.59
ICU length of stay, days	17 (11‒24)	17 (11‒24)	18 (10‒25)	0.98
Hospital length of stay, days	19 (11‒30)	20 (12‒31)	19 (11‒27)	0.39
Reintubation, n (%)	23 (10.3)	16 (10.1)	7 (11.5)	0.73
Tracheotomy, n (%)	46 (20.6)	34 (21)	12 (19.7)	0.87
In-hospital mortality, n (%)	188 (84.3)	133 (82.1)	55 (90.2)	0.14

Data are presented as average and interquartile range (25%‒75%) or the number of subjects and percentage, in parenthesis.

PEEP, Positive End-Expiratory Pressure; FiO_2_, The fraction of inspired oxygen; RR, Respiratory Rate; Pplat, Plateau Pressure of the respiratory system; Cst, Static Compliance of the respiratory system; Ph, Potential Hydrogen; PaO_2_, Partial Pressure of Arterial Oxygen; PaCO_2_, Partial Pressure of Arterial Carbon Dioxide; HCO_3_, Bicarbonate; PaO_2_/FiO_2_, The difference between the initial and final ratio of arterial oxygen partial pressure (PaO_2_: in mmHg) to fractional inspired oxygen (FiO_2_); Δ PCO_2_, The difference between the initial and final PaCO_2_; ACO, Anticoagulants; IMV, Invasive Mechanical Ventilation; ICU, Intensive Care Unit.

The *non-responders* group presented with more complications, leading to an interruption in the prone position. The most common complications include reduced oxygen saturation, unplanned extubation, hemodynamic instability, acute arrhythmia, and cardiac arrest. Furthermore, most patients received anticoagulants and vasopressors during treatment.

Although the *non-responders* group was placed in a prone position after the first 48h, there was no statistical difference between the two groups. On average, the *non-responders* group was placed in the prone position for at least 16h in 2 (1−3) sessions. The duration of the prone position and number of cycles did not differ between the two groups.

Similarly, no differences were observed between the duration of mechanical ventilation (in days), length of hospital or ICU stay (in days), and reintubation index. The in-hospital mortality rate was higher in the *non-responders* than *responders* group (90.2% vs. 82.1%), but the difference was not significant.

### Oxygenation improvement

A logistic regression analysis was performed to evaluate the factors associated with oxygenation improvement. Independent variables included age, sex, lung impairment observed on chest CT, previous immunosuppression, lung disease, SAPS III score, the total number of prone sessions, time taken to the first prone session, total duration of prone, D-dimer value, respiratory Cst, and occurrence of complications. Only SAPS III and complication rates were associated with improved oxygenation (Table 3). However, logistic regression analysis showed that only the SAPS III score predicted a better response to oxygenation after prone positioning (OR = 0.97 [0.94−0.99; p = 0.02]).

**Table 3:** Predictors factors of oxygenation improvement and mortality.

Variables	OR	CI (5%)	CI (95%)	p-value
**Oxygenation improvement**				
SAPS III	0.97	0.94	0.99	0.02
**Mortality rate**				
Male sex	0.21	0.06	0.70	0.01

Data from the logistic regression model. OR, Odds Ratio; CI, Confidence Interval; SAPS III, Simplified Acute Physiology Score.

### Mortality rate

The mortality rate analysis was performed using stepwise forward logistic regression, which includes age, sex, pulmonary impairment, previous immunosuppression, time until the first prone session, respiratory Cst, occurrence of complications, D-dimer value, and baseline pH. Variables associated with mortality were sex, previous immunosuppression, and respiratory Cst. In a logistic regression analysis, the male sex was a significant variable associated with worse mortality risk (OR = 0.21 [0.06−0.70; p = 0.01]), (Table 3).

## Discussion

This retrospective multicenter cohort study involved many elderly patients who underwent prone positioning due to a diagnosis of severe COVID-19-ARDS. The *non-responders* group (< 20-point in PaO_2_/FiO_2_) had higher SAPS III and SOFA scores than the *responders* group. In addition, the *non-responders* group had a higher incidence of complications; however, there was no difference in mortality rate. Finally, the authors found that a lower SAPS III score was a predictor of the response to oxygenation after the first prone session and that the male sex was a predictor of mortality risk.

It is well known that the elderly are disproportionately affected by COVID-19, representing a higher risk of infection and a severe form of the disease.^7^ Additionally, the elderly with a greater number of comorbidities may be more susceptible to mortality.^7^ However, the authors identified a gap in the literature that clarifies the response of this elderly group when subjected to prone positioning. Brazier et al. (2021) proposed a prone protocol model for elderly patients diagnosed with ARDS and respiratory failure; but their patients were not intubated, and the study did not assess their response to prone positioning.^16^ Therefore, to the best of our knowledge, this is the first study to analyze the effect of prone positioning in elderly patients intubated due to COVID-19-ARDS.

Reports indicate that COVID-19 is more prevalent among the male sex. Men are likely to be more severely affected, required to stay longer in the Intensive Care Unit (ICU), and present a greater fatality rate.^19^ Furthermore, older men are predisposed to a higher prevalence of metabolic syndrome, obesity, diabetes mellitus, and other chronic diseases.^19^ These results support our data suggesting that the female sex had a protective effect on mortality.

The present study identified that the *responders* group was more prevalent, but this response did not significantly reduce mortality. The mortality rate was 82.1% and 90.2% for the *responders* and *non-responders* groups, respectively. This may have occurred because the patients were older, had more comorbidities, and had high SAPS III and SOFA scores at admission to ICU.

The authors did not observe a difference in anthropometric data such as comorbidities or D-dimer levels, between the two groups. In general, the patients had high D-dimer levels and low lung compliance. The D-dimer is considered to play an important role in hypoxemia in ARDS. Consistent data show that the involvement of the pulmonary system in patients with COVID-19 has distinct characteristics and may cause endothelial damage. Furthermore, the combination of a high concentration of D-dimer and low lung compliance may dramatically increase the risk of mortality.^20^ This may also have affected the high mortality observed in the present study.

In contrast, the authors observed that SAPS III, a scale that includes the number of comorbidities and the initial patient's clinical condition, was a predictor of oxygenation response. The elderly in developing countries generally present a high number of comorbidities, mainly in males. These results suggest that better responses to the prone position occur when a patient has fewer comorbidities than a specific one (Table 1).

The present study suggests that each unit of SAPS III presents a probability of increasing by 0.03% in oxygenation. Thus, the authors can say that a difference of 20 points in SAPS III represents a 31% chance of response to prone positioning. Although no significant difference was found between improved oxygenation and mortality, a previous study showed that SAPS III and age predict mortality factors among ICU hospitalized COVID-19 patients.^21^ In addition, a Brazilian study concluded that patients with SAPS III value greater than 57 had higher mortality rates.^22^

The authors suggest that the high mortality observed in both groups may be associated with poor socioeconomic status. Evidence suggests that patients with COVID-19 had a higher mortality rate in a socially and economically disadvantaged region of New York, with a mortality rate over 75%.^23^ The previous conditions observed in the patients can be explained by the population in the developing country. In Brazil, even a population including younger patients with COVID-19 had poor results. The group showed a mortality rate of 69.3%.^24^ The study was part of the current study, including intubated patients, undergoing a prone positioning due to COVID-19-related ARDS. On the other hand, a similar study conducted by the Rush University Medical Center, presented a mortality rate of 21.4%.^25^ The general patient's age appears to be not different in both groups (59 [49‒69] and 58.5 [51.8‒69.3], respectively), but the mean SOFA score was higher in the studied group (9 vs. 6.8), strengthening the authors’ hypothesis.

The present study has several limitations. First, the main limitation of this study was missing data from the electronic medical records. Second, as opposed to a clinical trial, the decision and timing of prone positioning cannot be controlled in an observational study. This can lead to selection bias. Finally, the criteria used to assess the response to prone positioning are not universal; therefore, comparisons with other studies should be performed with caution. Despite these limitations, to our knowledge, this is the first multicenter cohort study to verify the response of patients with COVID-19-ARDS in an elderly group, which raises some hypotheses about the interventions of this group. However, prospective studies are required to better understand the response to the prone position in elderly patients affected by COVID-19-ARDS.

## Conclusion

The present study suggests that the oxygenation response to prone positioning in elderly patients with severe COVID-19-ARDS may correlate with the SAPS III score. Male sex may also be a risk predictor of mortality.

## Authors' contributions

All authors contributed to the study design, data collection, and manuscript revision.

## Funding

The study was supported by grant 312279/2018-3 from the Ministério da Ciência, Tecnologia e Inovação ‒ Conselho Nacional de Pesquisa (CNPq) and by the Coordenação de Aperfeiçoamento de Pessoal de Nível Superior – Brasil (CAPES) – Finance Code 001.

## Conflicts of interest

The authors declare no conflicts of interest.
